# American Medical Association

**Published:** 1852-07

**Authors:** 


*American Medical Association.*—We copy from the *Stethoscope* some particulars of the late meeting of this body not given in our last number. The following are the resolutions offered by Dr. Drake to which reference was made. It will be remembered that they were not adopted.

1. *Resolved*, That every report on a medical or other scientific subject shall be referred to a select committee, to be read, analyzed and reported on to the association; said select committee indicating its general character and worthiness of publication, provided the authors of every report shall have the right of appealing to the association.

2. *Resolved*, That no report shall be read before the association until it has been examined and reported on by the committee to which it may be referred; nor then but under an order of the association.

3. *Resolved*, That no report shall be published in the Transactions of the association but in virtue of its order.

4. *Resolved*, That all professional and other scientific communications made to the association, shall be referred and treated like the reports of committees.

5. *Resolved*, That the president, vice presidents and secretaries of the association shall be charged with the appointment of the aforesaid committees, being themselves eligible for such appointments.

6. *Resolved*, That the authors of all reports and papers aforesaid shall have the privilege of reading and explaining the same before the committees.

The report of the committee on the Medical Botany of the U. S. for 1850–'51, from Dr. A. Clapp, chairman, was presented and referred to the committee of publication.

THURSDAY, May 6, 1852.

The association was called to order at 9½ o'clock—Dr. Wellford, president, in the chair.

The minutes were read, amended and approved.

Dr. Green, of N. Y., offered the following resolutions, which were adopted:

1. *Resolved*, That at all future meetings of this association all reports of committees and all contributions on scientific subjects occupying more than ten pages of quarto post manuscript, be accompanied each by an abstract or synopsis embracing the principal points of such report or paper, which abstract or synopsis may be read before the association.

2. *Resolved*, That the above resolution be transmitted by the secretary to the chairman of each scientific committee.

Dr. Sutton, of Ky., moved that a committee of three be appointed, whose duty it shall be to inquire whether any, and if any, what action this association shall take in reference to requesting the Congress of the United States to have a large edition of the medical statistics, furnished by the census lately taken, published in a separate form for distribution among the medical profession of the United States, and to report to-day.

The chair announced the committee, to consist of Drs. Simons, of S. C., Boyle, of D. C., and Sumner, of Conn.

The report of the committee appointed to consider the various propositions which had been made, suggesting amendments to the constitution, being called for, the chairman, Dr. F. Campbell Stewart, of N. Y., read a report and resolutions, which Dr. Hays, of Pa., moved to refer to the committee of publication, with instructions to print.

Drs. J. K. Mitchell, of Pa., and W. Hooker, of Conn., discussed the merits of the report, when Dr. Lopez, of Alabama, raised a question of order as to the propriety of a discussion of the merits of the proposition.

The chair decided that the discussion was in order at this stage of the proceeding.

Dr. Lopez, of Ala., appealed from that decision, which appeal was not sustained.

The discussion was then continued at great length by many members.

During the discussion, the following replies were elicited from several gentlemen, by questions propounded by Dr. Watson, of New York:

From Dr. Horner, University of Pa.—The shortest term of medical study in the University required for the doctorate was three years, but that under some few and rare circumstances, a deviation had been permitted as an exception.

Drs. Davis and Rogers, of Virginia University, stated that their laws required no specified time: nine months, and eighteen years of age even, were sufficient, but that two years were generally devoted to the study of medicine by their graduates. They explained the course of instruction at the University at length.

Dr. Huston, of Jefferson Medical College, Philadelphia, said that three full years were required, but that occasions demanded sometimes a departure from the stringent rule.

Dr. Frost, from South Carolina, offered some interesting observations upon the much abused subject of medical education, and insisted that the profession had not retrograded. That there had been a steady and gradual improvement in our medical colleges generally, and brought to the notice of the association the attention which was observed in preparatory education in the medical college of South Carolina, which was highly creditable to the same. His remarks were listened to with attention, and brought forth observations of a like character from other members present.

The report and proposed amendments were received, after having been amended so as to read as follows: (We omit the report.)

ARTICLE I.—*Title of the Association.*

This institution shall be known and distinguished by the name and title of "The American Medical Association." It shall be composed of all the members of the medical profession of the United States of good standing, who acknowledge fealty to and adopt the code of ethics adopted by the association; and its business shall be conducted by their delegates or representatives, who shall be appointed annually in the manner prescribed in this constitution.

Strike out the whole of Article II, referring to "Members," and insert the following:

ARTICLE II.—*Of Delegates.*

Sec. 1. The delegates to the meetings of the association shall collectively represent and have cognizance of the common interests of the medical profession in every part of the United States, and shall hold their appointment from county, State and regularly chartered medical societies; from chartered medical colleges, hospitals and permanent voluntary medical associations in good standing with the profession. Delegates may also be received from the medical staffs of the United States army and navy.

Sec. 2. Each delegate shall hold his appointment for one year and until another is appointed to succeed him, and he shall be entitled to participate in all the business affairs of the association.

Sec. 3. The county, district, chartered and voluntary medical societies shall have the privilege of sending to the association one delegate for every ten of its resident members, and one more for every additional fraction of more than one half of this number.

Sec. 4. Every State society shall have the privilege of sending four delegates; and in those States in which county and district societies are not generally organized, in lieu of the privilege of sending four delegates, it shall be entitled to send one delegate for every ten of its regular members, and one more for every additional fraction of more than one half of this number.

Sec. 5. No medical society shall have the privilege of representation which does not require of its members an observance of the code of ethics of this association.

Sec. 6. The faculty of every chartered medical college acknowledging its fealty to the code of ethics of this association, shall have the privilege of sending one delegate to represent it in the association : *Provided*, That the said faculty shall comprise six professors, and give one course of instruction annually of not less than sixteen weeks on Anatomy, Materia Medica, Theory and Practice of Medicine, Theory and Practice of Surgery, Midwifery and Chemistry : *And provided also*, That the said faculty requires of its candidates for graduation—1st. That they shall be twenty-one of age ; 2d. That they shall have studied three entire years, two of which must have been with some respectable practitioner ; 3d. That they shall have attended two full courses of lectures, [not however to be embraced in the same year,] and one of which must have been in the institution granting the diploma, and also where students are required to continue their attendance on the lectures to the close of the session ; and 4th. That they shall show by examination that they are qualified to practice medicine.

Sec. 7. The medical faculty of the University of Virginia shall be entitled to representation in the association, notwithstanding that it has not six professors, and that it does not require three years of study from its pupils, but only so long as the present peculiar system of instruction and examination practised by that institution shall continue in force.

Sec. 8. All hospitals, the medical officers of which are in good standing with the profession, and which have accommodation for one hundred patients, shall be entitled to send one delegate to the association.

Sec. 9. Delegates representing the medical staffs of the United States army and navy shall be appointed by the chiefs of the army and navy medical bureaux. The number of delegates so appointed shall be four from the army medical officers and an equal number from the navy medical officers.

Sec. 10. No delegate shall be registered on the books of the association as representing more than one constituency.

Sec. 11. Every delegate elect, prior to the permanent organization of the annual meeting, and before voting on any question after the meeting has been organized, shall sign the constitution and inscribe his name and address in full, with the title of the institution which he represents.

FRIDAY, May 7, 1852.

The association was called to order by the president at 4½ o'clock, P. M.

The minutes of yesterday's session were read, amended and approved.

Dr. Atlee, of Pennsylvania, offered the following preamble and resolution, which was unanimously adopted:

Whereas it is the duty of patriotism to do homage to those who have been benefactors to their country; and whereas the medical profession in the United States, heretofore not wanting in patriotic feeling or action, desire to co-operate with the other public bodies and institutions of the country in rendering their profound reverence to the memory of him who was "first in peace, first in war, and first in the hearts of his countrymen:"

*Be it therefore resolved,* That a committee of five be appointed, whose duty it shall be to solicit subscriptions from members of the American Medical Association, for the purpose of procuring a suitable stone with an appropriate inscription, for insertion in the name of this association, into the national monument to the memory of Washington now in progress of erection at Washington city.

Dr. Wood, of Pa., then moved that instead of awarding five prizes of $50 each, annually, the association hereafter grant two prizes of $100 each, for the two best essays. Carried.

